# X-ray Computed Tomography for the Development of Ballistic Composite

**DOI:** 10.3390/ma13235566

**Published:** 2020-12-06

**Authors:** Grzegorz Ziółkowski, Joanna Pach, Dariusz Pyka, Tomasz Kurzynowski, Krzysztof Jamroziak

**Affiliations:** 1 Centre for Advanced Manufacturing Technologies/Fraunhofer Project Center (CAMT/FPC), Wroclaw University of Science and Technology, Lukasiewicza 5, 50-371 Wroclaw, Poland; tomasz.kurzynowski@pwr.edu.pl; 2 Department of Lightweight Elements Engineering, Foundry and Automation, Faculty of Mechanical Engineering, Wroclaw University of Science and Technology, Lukasiewicza 5, 50-371 Wroclaw, Poland; joanna.pach@pwr.edu.pl; 3 Department of Mechanics, Materials and Biomedical Engineering, Faculty of Mechanical Engineering, Wroclaw University of Science and Technology, Smoluchowskiego 25, 50-370 Wroclaw, Poland; dariusz.pyka@pwr.edu.pl (D.P.); krzysztof.jamroziak@pwr.edu.pl (K.J.)

**Keywords:** non-destructive testing, polymer–matrix composites (PMCs), impact behavior, finite element analysis (FEA)

## Abstract

This paper presents the results of research on ballistic panels made of polymer–matrix composites (PMCs). The analysis covers two types of composites produced by the authors based on high-density polyethylene (PEHD) and polypropylene (PP) reinforced with aramid fabric. Ballistic tests were carried out with the use of two types of projectile: 0.38 Special, and 9 × 19 Parabellum, which are characterized by different velocity and projectile energy. The study presents the X-ray computed tomography (XCT) analysis for structure assessment of ballistic panels and its impact behavior, further compared to the results of computer simulations conducted using the numerical analysis. The quality of the manufactured panels and their damage caused by a ballistic impact was assessed using a multi-scale geometry reconstruction. The mesoscale XCT allowed the internal composite geometry to be analyzed, as well as a unit cell of the representative volume element (RVE) model to be built. The RVE model was applied for homogenization and finite element (FEA) simulation of projectile penetration through the ballistic panel. The macroscale XCT investigation allowed for the quantitative description of the projectile’s impact on the degree of delamination and deformation of the panels’ geometry.

## 1. Introduction

The continuous development of composite materials allows for the improvement of the solutions used to protect people against the undesirable effects of weapons. The authors of numerous works have undertaken research on the materials used in ballistic panels, including the analysis of the type of composite reinforcement [1,2], the type of matrix [3], the material’s thickness [4,5,6,7], and the geometry and the method (sequence) of layering the fabrics (lay-up sequence) and connections of the reinforcement with the matrix [8,9,10]. The authors also deal with the development of the mathematical description of this issue [11,12,13,14]. Factors such as the shape, mass, structure, and kinetic energy of the projectile affect the resistance of ballistic panels, and therefore, a lot of attention is paid to experimental research that allows the impact of ballistic stroke on the nature of perforation/penetration [15,16,17] and damage [18] to be assessed. Aramid fiber-reinforced composites have a number of properties that make them the perfect choice for ballistic panels. They have low curb weight, high specific strength, and a high ability to absorb ballistic impact energy. These composites are used to make vests [19] and helmets [20,21], as well as light covers of military and civilian vehicles [22,23]. An important factor determining the behavior of a composite during impact is its matrix. Chemo and thermoset polymer matrices in aramid composites are characterized by high stiffness, and what is more, they brittle during impact. Thermoplastic polymers can be an interesting alternative to thermosets. As shown by the researchers of works [24], laminates that include thermoplastics as the matrix in composites intended for ballistic panels are a good solution due to, among others, the maximum deformation and pull-outs as a result of the weak adhesion between the fibers and matrix [24], as well as the increased dissipation energy as a result of delamination and debonding [24]. Therefore, an interesting direction of research is the assessment of the suitability of materials for ballistic panels with a matrix of high-density polyethylene (PEHD) or polypropylene (PP) reinforced with aramid fabric. Both of these polymers are cheap, melt easily, and are able to stick to aramid fibers.

**Figure 1:**
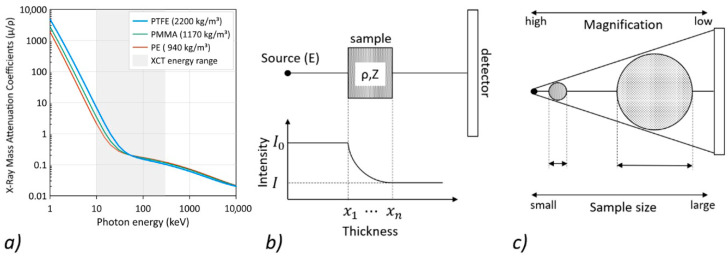
Factors affecting XCT scanning: (**a**) characteristics of X-ray absorption [35], (**b**) principles of XCT operation, (**c**) limitations related to the geometry of the measuring system and the sample size.

XCT is a non-destructive measurement method that has been known for a couple of decades. It uses X-ray radiation to obtain information about the internal and external structure of physical objects. Computed tomography is used in non-destructive analysis, materials science, metrology, and quality control. Among the methods widely used in assessing the impact behavior of composites, laboratory XCT tomography systems with a micro or nano-focus X-ray tube are becoming more and more popular [25]. The use of XCT enables the mapping of the three-dimensional geometry of fiber-reinforced polymer structures [25], damage propagation [26,27,28,29,30,31,32], the delamination of composites [31], or the damage of the panels after penetration by a projectile [33]. Due to the selective absorption of X-rays in matter, the geometry of a composite can be subject to qualitative characteristics [34,35]. In terms of the energy used in XCT, an attenuation contrast image is the result of photoelectric absorption (p), Compton scattering (C)-incoherent, and Raleigh scattering (R)-coherent. The linear attenuation coefficient (µ) can therefore be expressed by (1):

(1) $$\mu =\mu_{p}+\mu_{C}+\mu_{R}$$

and the dependence representing the mass attenuation coefficient can be presented as: (2) [36]:

(2) $$\frac{\mu }{\rho }=K\cdot \frac{Z^{m}}{E^{n}}$$

where *K* is the constant, *E* is the photon energy, ρ is the density, and *Z* is the atomic number. The values of *m* and *n* range from 3.8 to 2.0 and 3.2 to 1.9 for photoelectric absorption and Raleigh scattering, respectively. Compton scattering, however, depends only on the X-ray energy and is essentially independent of the atomic number *Z* [37]. The relationship between the X-ray mass attenuation coefficients (µ/ρ) and the photon energy for selected low-Z materials is shown in Figure 1a.

For simplicity, it can be assumed that the two main phenomena of X-ray interaction with matter dominate in the given energy window: the photoelectric effect (absorption) and incoherent scattering (Compton effect) [38]. The lower the photon’s energy, the more the photoelectric effect dominates. Moreover, the expected contrast will be due to the difference in density and composition of the material (Z numbers). In the case of PMCs, the atomic number is low, and therefore, the effect of increased contrast between the matrix and the fabric should be clear for low photon energy. Increasing the photon’s energy increases the dominance of the Compton scattering, and thus, XCT delivers information that is nearly proportional to ρ without information about the composition of the material. However, it is worth noting that the use of higher photon energies reduces the differences between the µ, in turn reducing the contrast between the imaged objects (Figure 1a). On the other hand, a high enough photon energy is necessary to obtain sufficient X-ray transmission through objects of a certain thickness (Figure 1b). The sample size significantly influences the achievable magnification, and therefore also the measurement resolution, as shown in Figure 1c. For this reason, it is difficult to achieve a high-contrast resolution for large sample sizes. Despite the above-mentioned limitations, the volumetric data obtained on the basis of the XCT reconstruction provide the necessary information to design realistic multiscale models for use in numerical simulations [39]. XCT mesoscale measurements enable representative volumetric models (RVE) with a fabric and matrix to be extracted, which in turn allows for the simulation of composites with defects arising during their formation [40]. The quantitative analysis of internal damage in deformed composite panels based on XCT data was proposed in [41], while in [42], the possibilities of using XCT data in supporting the process of creating numerical models and their validation were presented.

The process of designing and optimizing protection material systems, as opposed to the classical approach that relies heavily on a design-make-shoot iterative process, has currently been replaced by rapid iterations of modeling and simulation (with ballistic evaluation used selectively to verify satisfactory designs). The aim of the new paradigm of developing lightweight protection material is to enable, through characterizations, microstructures, behaviors, and deformation mechanisms, the design of superior protection materials, as well as to accelerate their implementation in armor systems [43]. Therefore, there is a need to develop new concepts of multi-scale evaluation and modeling techniques for predicting energy dissipation mechanisms, as well as for the quantitative verification of simulation results that allow for rapid iterations of modeling.

The aim of this work is to present an XCT based approach for assisting both the design process of lightweight protection material and also the quantitative evaluation of the impact behavior and numerical simulation accuracy of PMC ballistic panels. Two types of composites based on PP and PEHDmatrices were selected for the quantitative description of the projectile impact phenomena. Ballistic tests were conducted using two types of projectile: 0.38 Special and 9 × 19 Parabellum, which obtain different velocities and projectile energies. Basic information on the limitations and possibilities of using XCT to evaluate the geometry of the PMCs is presented, with particular emphasis on the influence of the X-ray voltage source on the contrast composite reconstruction. Based on the mesoscale volumetric XCT data, the composite RVE model was designed and a numerical simulation was performed. The mesoscale XCT investigation allowed for the quantitative description of the projectile impact on the degree of the delamination and deformation of the panel’s geometry. Currently, the geometric data of composites obtained as a result of simulations are compared with the XCT data in a mainly qualitative manner, e.g., by comparing the simulation results and the XCT cross-section [17,42]. Therefore, the new method proposed in this paper of validating simulation results based on the analysis of ballistic panel geometry deviations may increase the accuracy of numerical models and allow for rapid iterations of PMC modeling.

## 2. Materials and Methods

### 2.1. Sample Preparation

The PMC composites described in this paper were produced by PP HP548R and high-density PEHD Hostalen ACP9255 Plus manufactured by LyondellBasell (Rotterdam, The Netherlands). The laminates were produced using the aramid fabric Twaron^®^1210 dtex weight of 173 g/m^2^ and the plain weave (warp yarns: Aramid 1210 dtex Type 2200 and weft fibers: Aramid 1210 dtex Type 2200). The tensile yield of the aramid fabric is 1140 (N/cm) determined according to ISO 13934-1: 2000, thickness 250 μm ISO 5084: 1998 (data according to the producer’s data sheet) [44]. The laminates were formed in two stages. The first stage consisted of pressing the polymers in the form of granules until they obtained the shape of tiles with dimensions of 100 mm × 100 mm and an average thickness of 0.5 mm. PP and PEHD were heated for 2 min at temperatures of 230 °C, and for another 2 min at temperatures of 190 °C with a pressure of 2 MPa. Then, the prepared polymer plates were placed alternately with the aramid fabric into a metal mold, and then pressed under a pressure of 6 MPa until the samples got cooled [44]. The characteristics of the produced ballistic laminates are summarized in Table 1.

**Figure 2:**
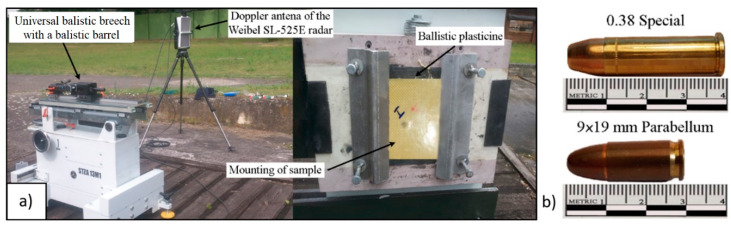
Diagram of the test stand: (**a**) ballistic barrel with accessories and a support for specimen mounting, (**b**) projectile used in the ballistic tests.

The 0.38 Special S&B and 9 mm × 19 mm Parabellum MESCO projectiles were used in this study. The ballistic panels were overshot (at an angle of 90° and distance of 5 m) from the PROTOTYPE ballistic barrel (Bareel cal. 0.38 Special, length 250 mm, and Barrel cal. 9 mm Browning court, length 250 mm). Figure 2a shows the stand used to fire the samples.

### 2.2. Ballistic Tests

The 0.38 Special S&B and 9 mm × 19 mm Parabellum MESCO projectiles were used in this study. The ballistic panels were overshot (at an angle of 90° and distance of 5 m) from the PROTOTYPE ballistic barrel (Bareel cal. 0.38 Special, length 250 mm, and Barrel cal. 9 mm Browning court, length 250 mm). Figure 2b shows the projectile used to fire the samples.

The muzzle velocity of the projectile was measured using the Doppler Weibel SL-525E radar.(Alleroed, Denmark) The speed measurement was made at a distance of 5 m from the sample. The test parameters are summarized in Table 2.

In order to verify the ballistic resistance of the panels prepared, three types of research were performed. First, in the PEHD_S test, panels made of PEHD were fired upon with 0.38 caliber projectile. Due to the fact that the panels in the PEHD_S test did not stop the 0.38 caliber projectile, the PEHD samples were not tested with a higher energy projectile (9 mm × 19 mm caliber). The second type of test (PP_S) consisted in overshooting panels made of PP with a 0.38 caliber projectile. Because the 0.38 caliber projectile did not penetrate the PP panel, in the third test (PP_P), the panel was tested with higher energy projectile (caliber 9 × 19). The tests were repeated three times for each of the research series.

### 2.3. X-ray Computed Tomography

The reconstruction of the composite panels was performed using the XCT Metrotom 1500 system (Carl Zeiss, Oberkohen, Germany) with a micro-focus 225 kV X-ray tube. The system is equipped with a 16-bit detector with dimensions of 40 cm × 40 cm and a resolution of 1024 × 1024 pixels. The distance between the lamp and the detector is 1501 mm, and the maximum possible resolution (voxel size) is 7 µm. Prior to each measurement, the XCT system was calibrated for scale error correction according to the manufacturer’s procedure. Observations of the entire panels were carried out with a resolution corresponding to the voxel size at the level of 135.27 µm. The obtained resolution was related to the size of the scanned panels. The percentage of the delamination volume was determined using the region growing algorithm according to the relationship (3):

(3) $$D=\frac{V_{d}}{V_{m}+V_{d}}\times 100\%$$

where $V_{d}$—delamination volume and $V_{m}$—material volume.

The influence of the X-ray tube voltage on the contrast was tested for reconstructions with a resolution of 30.00 µm for a selected fragment of the PP composite. The absorption contrast between the aramid fibers and the polymer matrix can be calculated using the relationship [29] (4):

(4) $$\mathrm{Contrast}=\frac{\mu_{Fibres}-\mu_{Matrix}}{\mu_{Matrix}}$$

where µ_*Fibers* is the maximum value of the grayscale for aramid fibers, and µ_*Matrix* is the mean value of the polymer surrounding the fibers. In order to quantify image quality differences, contrast-to-noise ratios (CNR) were calculated as (5):

(5) $$\mathrm{CNR}=\frac{\left|\mu_{Fibres}-\mu_{Matrix}\right|}{\bar{\sigma }}$$

where $\bar{\sigma }$ is the image noise specified as the standard deviation for the background.

In order to compare the internal structures of the PEHD and PP composites with a high resolution, the ROI with dimensions of 5 mm × 5 mm were reconstructed with the voxel size of 10.60 µm. A local thresholding method was used to extract three-dimensional geometric data from two-dimensional tomographic sections. Data visualization and porosity analysis were performed using VG Studio MAX 2.0 software (Volume Graphics GmbH, Heidelberg, Germany).

### 2.4. Numerical Simulation

**Figure 3:**
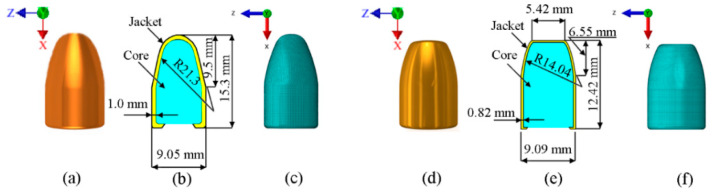
Projectile geometry: (**a**) 9 mm Parabellum, (**b**) dimensions, (**c**) discrete model, (**d**) 0.38 Special, (**e**) dimensions, (**f**) discrete model.

Aramid fabric on a polymer matrix were modeled as a layered element with the separation of individual layers. After preliminary simulations, the projectile shell modeling was later abandoned due to negligible participation in the ongoing process, which was due to the fact that the projectile core plays a decisive role here. The dimensions of the shells are shown in Figure 3.

It was also noted that the rotational speed of the projectile as a component of the total impact energy had a slight impact on the destruction of the panels, and this was therefore also omitted in the calculations. A constitutive equation with Johnson–Cook destruction was used for all the models [45,46]. This model is the one most used in impact analysis (6):

(6) $$\sigma_{y}=\left(A+B\varepsilon_{p}^{n}\right)\times {\left(1+B{\dot{\varepsilon }}^{*}\right)}^{C}$$

where $\varepsilon_{p}$ is the equivalent plastic strain, and ${\dot{\varepsilon }}^{*}$ is the normalized effective plastic strain rate defined as $\left(\frac{{\dot{\varepsilon }}_{p}}{{\dot{\varepsilon }}_{0}}\right)$. The material constants are *A*, *B*, *n*, and *C*. Destruction is represented by the following Formula (7):

(7) $$\varepsilon_{f}=\left(D_{1}+D_{2}e^{D_{3}\sigma^{*}}\right)$$

where $D_{1}$, $D_{2}$, $D_{3}$, and $D_{4}$ are material parameters, *σ* * is the dimensionless ratio expressed as the pressure *P*, and *σ* is the effective stress (von Mises equivalent stress). Table 3 summarizes the parameters of the analyzed laminates, and Table 4 shows the material characteristics of the projectile.

**Figure 4:**
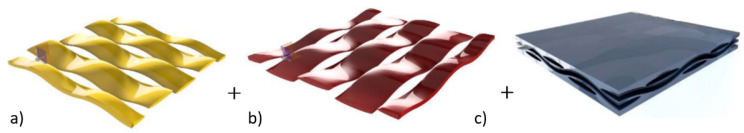
Projectile geometry: (**a**) 9 mm Parabellum, (**b**) dimensions, (**c**) discrete model.

The numerical model was implemented in the ABAQUS environment. To calculate the failure criterion, the modified multi continuum theory (MCT) method was used with the changed limit strain criterion *ε_gr_*. Geometric models of the reinforcement (aramid fabric) were developed based on geometry registered using XCT examination. The directional orientation of the transverse fibers (Figure 4a) and longitudinal fibers (Figure 4b) was performed, thus obtaining the model of the cross-linking matrix (Figure 4c).

The boundary conditions were imposed by fixing the panel by blocking translation and rotation in the x, y, and z axes. The type “hard contact” between the layers was set. The initial speed of the projectile was set at 370 m/s. It was also assumed that destruction occurs in the panel when the elongation of 2% is exceeded. The finite element analysis and smooth particle hydrodynamics (FEA)/(SPH) hybrid method was adopted in the presented simulation [48,49,50]. The entire model, both the projectile and the panel under consideration, were designed as volumetric elements with applied discretization (FEA). After exceeding the limit deformation *ε_gr_* of the individual elements, the conversion into a material point described by the free point equations of the SPH method took place.

## 3. Results

### 3.1. Energy-Dependent XCT Contrast

Due to the large number of factors affecting the quality of XCT imaging, it becomes necessary to indicate the significant impact of changes in the X-ray tube voltage on the contrast difference for objects of similar density, such as PMCs. In order to quantify the effect of the applied X-ray tube voltage, the same area of the PP composite sample was reconstructed four times with the measurement parameters listed in Table 5. The current of the X-ray tube is chosen in a way that the tube power was each time the same. This allowed the resolution to be unchanged for each of the measurements, i.e., the relation between the X-ray tube’s focal spot and voxel size remained constant in each case. Thanks to this, the blurring of the image remained the same [51].

**Figure 5:**
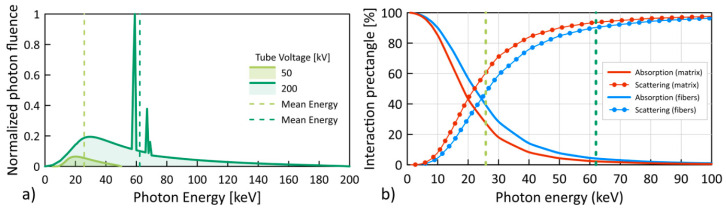
X-ray spectra for tube voltages 50 and 200 kV [52]: (**a**,**b**) the percentage of absorption (photoelectric effect) and scattering (incoherent scattering) in the PP matrix and aramid fibers [36]. Vertical lines show the mean energies of the spectrum.

The laboratory XCT systems generate bremsstrahlung continuous spectra, and the tube voltage refers to the maximum voltage applied across an X-ray tube during the creation of X-rays within it. Demonstration of the effect of spectrum energy on XCT contrast can be approximated by using the value of the mean energy spectrum (Figure 5a), which can be estimated, e.g., with Spekcalc software using the deterministic model [52]. On the other hand, the absorption and scattering of radiation for the composite matrix and reinforcement can be presented as a percentage of these phenomena with regards to the photon energy (Figure 5b).

The relations presented in this way allow the influence of the spectrum energy on the X-ray absorption and scattering characteristic to be understood. For example, the mean spectrum energy corresponding to the applied minimum (50 kV) and maximum (200 kV) voltages of the X-ray tube is marked as green vertical lines in Figure 5a. For minimum voltage of the X-ray tube, the mean spectrum energy is 25.8 keV, which corresponds to the difference in absorption and scattering of radiation for the two selected materials at the level of about 10% (Figure 5b). On the other hand, for the maximum voltage of the X-ray tube, the mean spectrum energy is 62.3 keV, which corresponds to the difference in absorption and scattering of radiation at the level of about 2–3%. Thus, the XCT reconstruction recorded with the lowest voltage should have the highest contrast.

**Figure 6:**
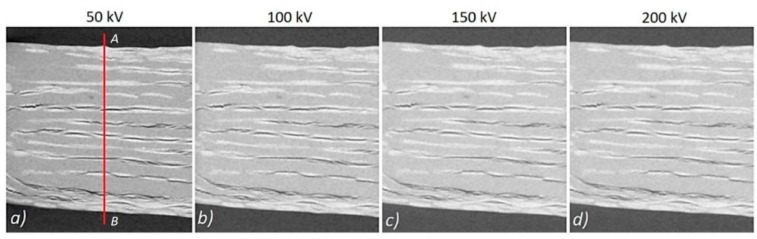
Cross-sections through the PP composite obtained with the X-ray tube voltage: (**a**) 50 kV, (**b**) 100 kV, (**c**) 150 kV, (**d**) 200 kV.

This is confirmed by the results of the experiment, which are shown in the form of 2D cross sections through the sample in Figure 6. There is a clear change in the contrast between the reconstruction with the lowest voltage (Figure 6a) and the other values (Figure 6b–d).

**Figure 7:**
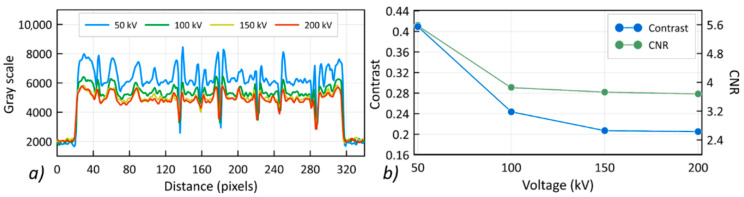
The influence of the X-ray tube voltage on the contrast recorded for the PP composite, (**a**) change of contrast along the A-B line, (**b**) change of contrast and contrast-to-noise ratio (CNR).

Profiles showing the change in contrast along the A–B line (Figure 6a) for the different applied voltages of the X-ray tube are shown in Figure 7a. The recorded grayscale value was significantly reduced for the high voltage reconstruction. Figure 7b shows the change in the values of contrast and contrast-to-noise ratio (CNR) with an increasing voltage of the X-ray tube. A decrease in contrast of around 50% was recorded for the 200 kV reconstruction compared to that of the lower voltage of 50 kV, while the CNR value decreased by approximately 36%. This means that with the increase in voltage, the level of measurement noise decreased.

**Figure 8:**
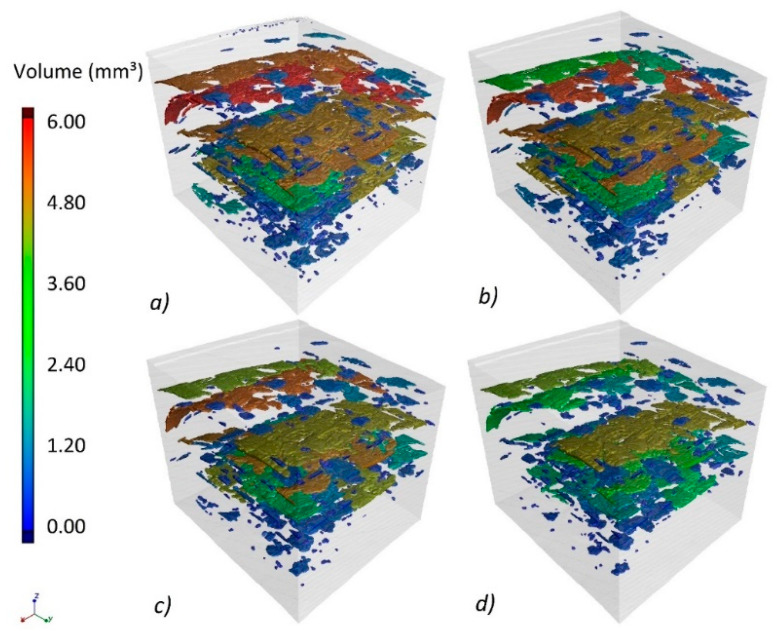
Change in the recorded volume for the reconstruction of the PP composite with different voltages of the X-ray tube, (**a**)50 kV, (**b**)100 kV, (**c**)150 kV, (**d**) 200 kV.

The consequence of deteriorating contrast is the reduction in the accuracy of the edge detection of the polymer matrix, fibers, and pores present in the composite. This can be seen in Figure 8, where the pore volume is registered for successive X-ray tube voltage levels in the form of a color map. With increasing tube voltage, the observed porosity decreases, and the number of pores with the smallest volume also decreases.

**Figure 9:**
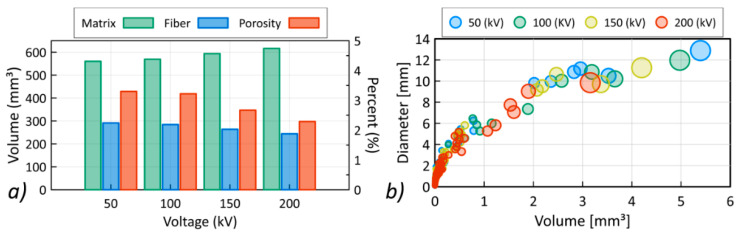
Volume change for the matrix, fibers, and porosity (**a**), change in volume and size of pores, (**b**) with increasing voltages of the X-ray tube.

The quantitative evaluation of changes in the volume of fibers, polymer matrix, and the porosity characteristics are shown in Figure 9a. As the voltage of the X-ray tube increased, an increase in the volume of the matrix by 9.98% was recorded. The volume of the fibers decreased by 16.15%, while the porosity value decreased by over 1%.

**Figure 10:**
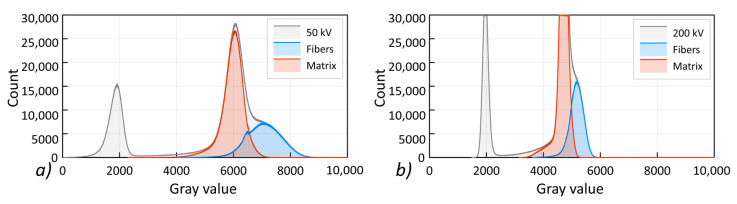
Grayscale histograms recorded for the PP reconstruction with X-ray tube voltage: (**a**) 50 kV, (**b**) 200 kV.

Figure 9b shows the relationship between the diameters of the registered pores and their volume. With an increasing voltage, both the size of the registered pores and their diameters decreased. The observed effect indicates the significant influence of the obtained contrast on the accuracy of determining the phase boundary present in the composite. The shape of the gray scale histograms obtained for the 50 and 200 kV reconstructions is shown in Figure 10.

Reducing the contrast increases the number of voxels in the area where the grayscale for the polymer matrix and the fibers overlaps. Therefore, the mapping of the shape of the composite components is less accurate for a reconstruction with a higher voltage and a higher mean X-ray beam energy.

### 3.2. XCT Analysis of PMCs in Mesoscale

**Figure 11:**
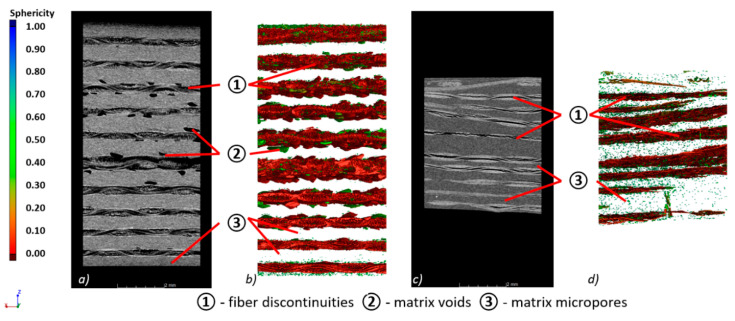
Internal structure of PMCs, (**a**) 2D view of the PEHD sample, (**b**) 3D view of the PEHD sample, (**c**) 2D view of the PP sample, (**d**) 3D view of the PP sample.

A comparison of the composites based on PEHD and PP matrix was carried out for fragments of panels (Figure 11). On the basis of the results obtained in the previous sections, the voltage of the X-ray tube was set at 50 kV, which guarantees a high contrast of the reconstruction. A magnification of 36.6× allowed for the mesoscale reconstruction and registration of the composites’ features, such as the arrangement of the aramid fibers forming the reinforcement, the determination of the matrix volume, or the discontinuities inside the composite (Figure 11).

**Figure 12:**
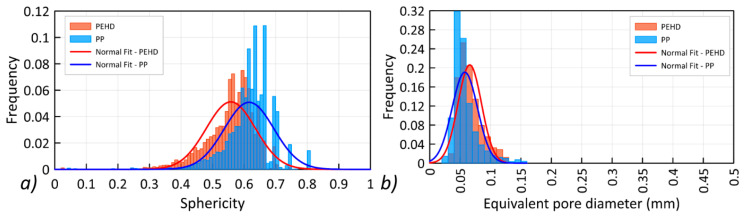
Characteristics of pores registered in the composite: (**a**) sphericity, (**b**) equivalent pore diameters.

Three types of defects were identified in the PMCs samples: fiber discontinuities, matrix voids, and matrix micropores. The PEHD matrix shown in Figure 11a was characterized by a large number of low sphericity discontinuities occurring in the spaces between the fibers (Figure 11b). In the PEHD matrix, a significant number of voids with large diameters and a relatively small number of micropores were observed. In the case of the PP composite, delaminations between the aramid fibers are clearly visible (Figure 11c,d); however, their volume is much smaller than that of the PEHD matrix. In this case, a significant number of micropores occurring in the entire volume of the matrix was recorded. The sphericity distribution of the registered defects is shown in Figure 12a, and the distribution of the smallest defect diameters in the tested samples is presented in Figure 12b.

The greater sphericity of the defects occurring in the PP sample (Figure 12a) and the smaller pore diameters (Figure 12b) clearly indicate the presence of a greater number of micropores in the PP matrix, which confirms previous conclusions. The obtained results indicate that the aramid fibers are insufficiently saturated with the polymer matrix in two analyzed cases; however, the degree of saturation for the PEHD matrix is much lower than for the PP matrix.

### 3.3. Ballistic Tests-XCT Evaluation of Panel Damage

**Figure 13:**
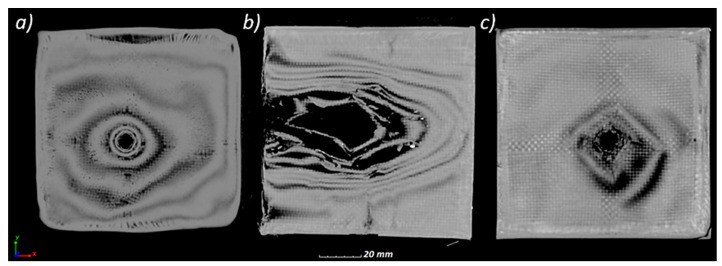
CT cross-sections through the composites after the tests: (**a**) PEHD_S, (**b**) PP_S, (**c**) PP_P.

In order to assess the delamination and deformation of the panels under the influence of ballistic impact, the geometry of the entire samples was reconstructed. To achieve a sufficient X-ray transmission through the entire geometry of the samples, the X-ray tube voltage was set at 130 kV during XCT scanning. The cross sections of the samples with visible delamination are shown in Figure 13. Increasing the X-ray tube voltage reduced the contrast between the aramid fibers and the matrix of the scanned composites. However, the applied resolution is sufficient to assess the degree of panel delamination.

**Figure 14:**
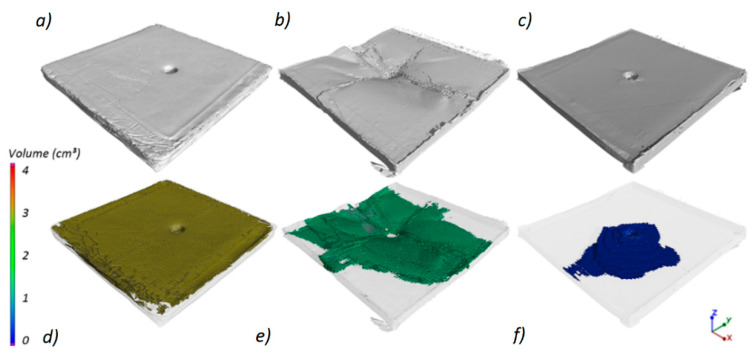
Three-dimensional view of delamination in the composites after testing: (**a**) PEHD_S (D = 22.26%), (**b**) PP_S (D = 5.89%), (**c**) PP_P (1.49%). Transparent view for samples after the tests: (**d**) PEHD_S (**e**) PP_S (**f**) PP_P.

In all cases, delamination started from the impact point and propagated throughout the panels. The quantitative analysis of the panels’ delamination after the ballistic tests is presented in Figure 14. The “0.38 Special” projectile with the lowest velocity penetrated the PEHD panel (Figure 14a). The degree of delamination caused by the projectile penetration is shown in Figure 14d, with the percentage volume of delamination being 22.26%. In the case of the PP panel, there was a significant geometry deformation with a partial delimitation (5.89%) of the panel (Figure 14b,e). The use of the “9 mm × 19 mm Parabellum” projectile with a higher velocity resulted in the perforation of the PP panel (Figure 14c). The percentage of delamination in this case was the lowest and amounted to 1.49% (Figure 14f).

**Figure 15:**
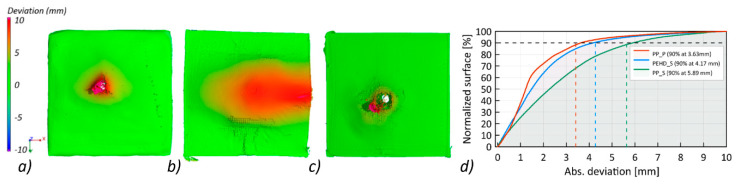
Comparison of surface deformation in the form of a color map after tests: (**a**) PEHD_S, (**b**) PP_S, (**c**) PP_P, and (**d**) cumulative histogram of surface deformation after ballistic impact for the tests.

In order to compare the deformations of the ballistic panels, the actual nominal comparison method was used. The fitting of models in the same coordinate system was carried out using the “best-fit” algorithm. The deviations showing surface deformations before and after the ballistic test were visualized in the form of a color map in Figure 15a–c.

The largest surface deformations were recorded for the PP_S panel, which stopped the projectile, where 90% of the surface was at a distance of 5.89 mm from the reference plane (Figure 15d). The smallest deviations were recorded for the PP_P panel, in which the projectile penetrated the shield and deformed 90% of the surface to a value of 3.63 mm.

### 3.4. FEA Results and XCT Verification

**Figure 16:**
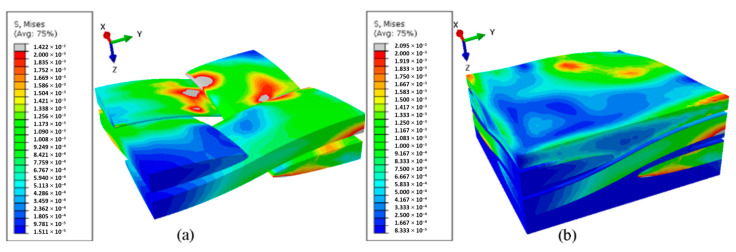
Representative volumetric models (RVE) unit cell load results: (**a**) a numerical model of the volume cell, (**b**) a geometric model of the elementary cell.

**Figure 17:**
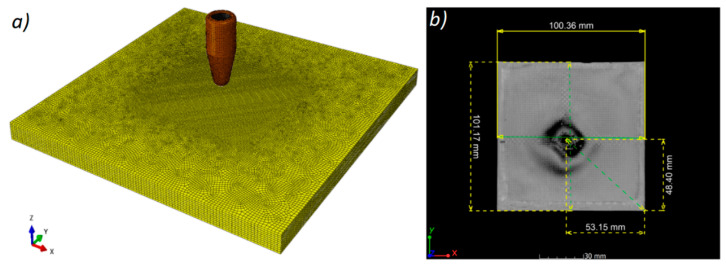
Discretization of the elements of a laminate board and a 9 mm × 19 mm projectile (**a**) and (**b**) boundary conditions for the projectile impact point determined on the basis of the XCT reconstruction.

The aim of the FEA experiment was to obtain a deformed geometry of the ballistic panel after penetration by the projectile. The simulation was carried out for the PP_P sample, in which the “9 × 19 Parabellum” projectile penetrated the panel on the PP matrix. In the presented results of the numerical analysis based on the RVE unit cell [50], uneven loads were observed (Figure 16). This was caused by the different load transfer by the materials, i.e., from typical stiffness characteristics. After the homogenization process, the fabric layers and the matrix were designed in the form of a plate with dimensions of 100 mm × 100 mm and a thickness of 0.5 mm (Figure 17). The projectile’s flight path relative to the panel was defined according to dimensions obtained experimentally (Figure 17a).

The discretization of the elements was performed at the level of 0.5 mm with the mesh density to 0.1 mm in the area of the projectile impact.

**Figure 18:**
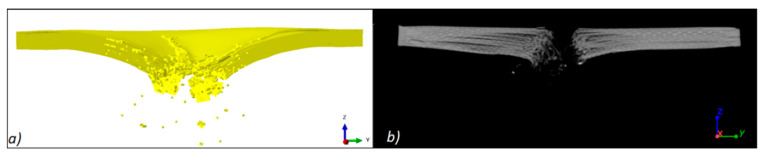
Cross-section of the panel after the projectile has passed: (**a**) simulation result, (**b**) experiment result.

Figure 18a shows the results of the simulation of the PP panel penetration by the “9 × 19 Parabellum” projectile. There is visible delamination of individual composite layers and elements of the SPH. Thanks to the use of the hybrid FEA/SPH method, the total mechanical energy in the system remains constant, which directly affects the obtained results.

**Figure 19:**
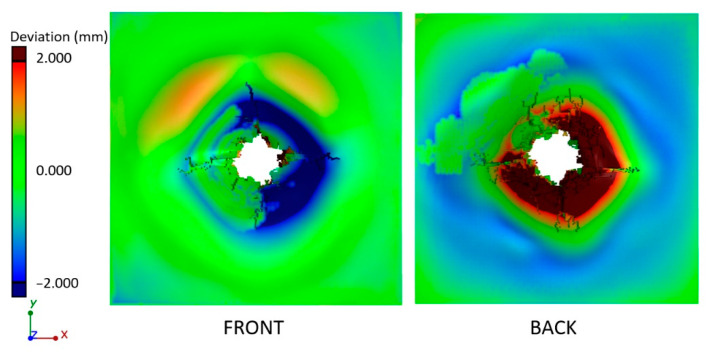
Actual/nominal comparision between the numerical model after deformation and the experiment results after deformation.

The analysis of geometry deviations after the simulation and ballistic experiment was carried out using the actual nominal comparison method. The model after simulation was exported from the ABAQUS environment and converted to a surface model (STL), and then fitted to the appropriate coordinate system. The results in the form of a deviation map plotted on the simulation model are shown in Figure 19.

**Figure 20:**
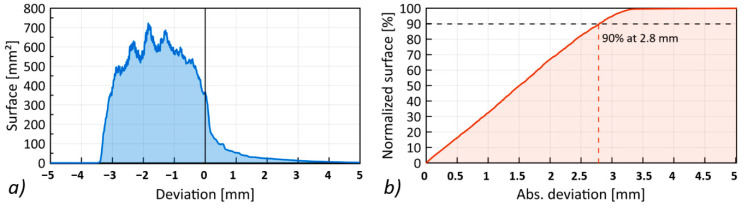
Quantitative evaluation of the differences in the simulation and experiment: (**a**) geometry deviation histogram, (**b**) cumulative deviation histogram.

The largest deviations exceeding +/− 2 mm were recorded around the point of penetration of the projectile. The back surface of the simulation model deforms more at a greater distance from the point of penetration of the projectile than in the experimental model. Greater deformation of the simulation model is visible in the form of the histogram presented in Figure 20a, where most of the deviations are negative. However, based on the cumulative histogram of deviations (Figure 20b), it can be concluded that 90% of the deviations of the compared surfaces are within a distance of 2.8 mm.

## 4. Discussion

Delamination and debonding were observed for all the analyzed ballistic panels. The analysis of the 3D images obtained for the panels after penetration raises the question of the reason for the significant delamination for PEHD_S and PP_S samples with a comparable initial energy of the projectile. This may be due to the lower interaction between the aramid fibers and the PEHD when compared to the PP. PEHD is a linear polymer with no backbone substituents, which may explain its low interaction with other compounds. Another factor that should be considered is the thickness of the composites. The PEHD panel is thicker than the PP panel, despite the same pressure being used in the manufacturing process. This may result from a different melt flow rate (MFR) for both composites, the value of which was determined in accordance with ISO 1133. The MFR for PP is 23 g/10 min (230 °C/2.16 kg), while for PEHD, it is 0.4 g/10 min (190 °C/5.0 kg). This index affects the degree of penetration of the fabric fibers through the matrix material during the composite production process. This also explains the greater degree of fiber discontinuities and the occurrence of voids in the PEHD matrix, as demonstrated in the previous section. As a result, a less homogeneous structure of the PEHD matrix, a greater number of defects, and a lower degree of saturation of the aramid fabric resulted in a lower effectiveness of the panel and its greater degree of delamination.

The degree of panel deformation results from the significant differences in the velocity of the projectiles, amounting to about 90 m/s. This difference significantly affects the material deformation rate, which is defined by the Cuniff parameter [48]. The effect of velocity causes the fibers to shear instead of stretch, which significantly reduces the extent of deformation due to the fibers being sheared faster. The shape of the projectile used was also different. The “9 × 19 Parabellum” projectile has a more favorable shape and aerodynamic drag coefficient when compared to the “0.38 Special” bullet for the composite destruction process. The influence of the geometric shape of the bullet on the penetration process has been described in more detail in the literature [49,53].

Deviations and differences obtained in the conducted analysis result from the adopted numerical method. This is due to the adopted composite model in the form of volumetric elements with the specification of individual layers as homogenized elements, the method of discretization of the elements (differences up to 5% depending on the adopted discretization elements), the peripheral restraint of the sample, and the setting of the initial velocity as averaged values of the measured projectile velocities during tests of whether the method of parameter homogenization itself may affect the accuracy of the results obtained. On the other hand, the method of verifying the results presented in this chapter, in the form of comparing the geometry after the experiment and simulation, is the basis for the optimization of the simulation process of complex dynamic phenomena, such as the interaction of a ballistic projectile with protective armor.

**Figure 21:**
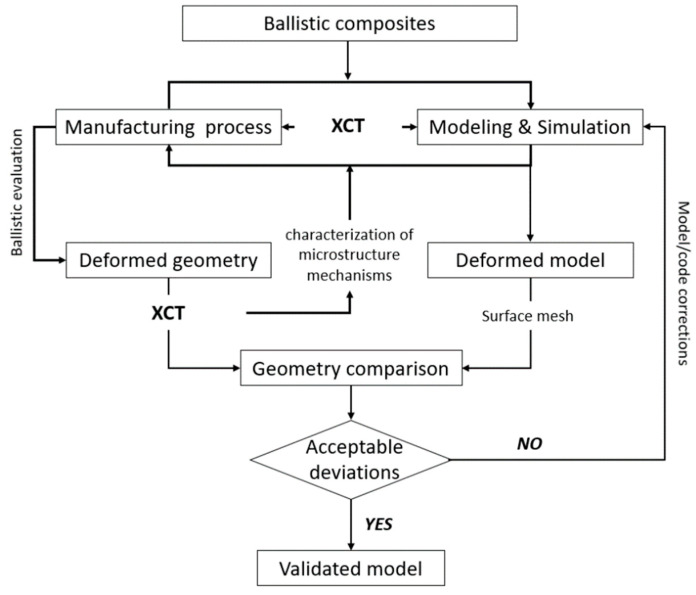
XCT approach to ballistic composite development.

The application of the XCT allows for quick iterations of designing numerical models, as well as fast verification of the results provided in the simulation tests. This in turn increases the flexibility and adaptability of designing protection materials and systems. XCT, thanks to its unique possibility of non-destructive inspection of objects at various resolution levels, provides the opportunity of analyzing and controlling the composite’s manufacturing processes and their testing procedures. It also supports numerical methods by converting the actual geometry to a digital model. A new area of application of XCT is the verification of numerical simulation results by comparing the geometry of a deformed sample after a ballistic projectile penetration with the deformed geometry after the simulation test (Figure 21).

The application of the XCT method for the development of PMC composites significantly speeds up the process of their production, the assessment of their ballistic resistance, and the verification of the geometric accuracy of numerical calculations, which include the phenomenon of the penetration of the bullet through the panel. One of the main problems with the XCT examination of PMC structures is the low contrast between the reinforcement and the matrix of composites, which reduces the accuracy of the obtained results. Strategies to increase the contrast of images obtained with composites include scanning with phase contrast or contrast agents [25], but these possibilities are not always available for laboratory XCT systems. Therefore, the use of lower voltages of the X-ray tube in the case of PMC scanning allows us to obtain higher contrast, increasing the accuracy of the imaged structures. The results presented in this paper show that the contrast can be increased by using lower X-ray tube voltages, thus increasing the accuracy of XCT testing of composites.

## 5. Conclusions

The paper presents the XCT approach for supporting the design and optimization of ballistic composites (PMCs). The most important conclusions from the experimental examinations and numerical simulations can be summarized as follows.

- The use of lower values of the X-ray tube accelerating voltages allows for a greater contrast in the XCT reconstruction of PMC structures to be obtained. A decrease in contrast of around 50% was recorded for the reconstruction with voltage at the level of 200 kV when compared to the reconstruction with 50 kV. This is due to the greater contribution of the photoelectric effect in the XCT image formation and better differentiation of the composition of the material.
- Reducing the reconstruction contrast of the PP composite resulted in a decrease in the registered aramid fibers’ volume by 16.15%, while the porosity value decreased by over 1%. At the same time, the volume of the matrix increased by 9.98%. This indicates that better accuracy of the XCT reconstruction is achieved when scanning composites with a lower X-ray tube acceleration voltage.
- Characterization of composites carried out in the mesoscale showed a greater number of defects and delamination recorded for the PEHD sample when compared to the PP sample. For the PEHD sample, a lower sphericity of pores was recorded with higher diameters and a higher level of delamination.
- The PP panel was characterized by better ballistic resistance than the PEHD panel, stopping the “0.38 Special” projectile with the energy of 392 J. The PP panel was penetrated by a “9 mm × 19 mm Parabellum” projectile with an energy of 548 J.
- The quantitative XCT comparison of the panel deformation after the ballistic test and FEA simulation showed that 90% of the deviations of the compared surfaces are within a distance of 2.8 mm. The deviations and differences obtained in the performed numerical analysis result from the adopted simulation method. Therefore, the aim of further research will be to optimize numerical methods in order to reduce geometry deviations between the results of the simulation and the experiment.

## Figures and Tables

**Table 1:** Types of manufactured ballistic panels.

Matrix	Layers	Thickness [mm]	Mass [g]
PEHD	10	9.0	77
PP		6.1	57

**Table 2:** Parameters of the ballistic test.

Type of Projectile	Caliber	Velocity [m/s]	Mass [g]	Energy [J]	Test Type
Special S&B	0.38	280 ± 4	10	392.0	PEHD_S
					PP_S
Parabellum MESCO	9 × 19	370 ± 8	8	547.6	PP_P

**Table 3:** Material properties of the panel [44,45,47].

Material	E	ν	ρ	A	B	n	C	D_1_	D_2_	D_3_
	[GPa]	[-]	[kg/m^3^]	[MPa]	[MPa]	[-]	[s^−1^]	[-]	[-]	[-]
Aramid fibers	71.0	0.36	1444	2450	2500	0.1	1.0	1.4	0.28	0.10
Polypropylene (PP)	1.5	0.45	900	26	10	0.15	0.1	0.068	5.382	−2.534
Polyethylene (PEHD)	0.7	0.46	960	40	25	0.18	0.1	0.068	5.382	−2.534

**Table 4:** Material properties of the projectile [44,47].

Projectile	E	ν	ρ	A	B	n	C
	[GPa]	[-]	[kg/m^3^]	[MPa]	[MPa]	[-]	[s^−1^]
Core: Lead (alloy of PB1 and antimony)	1.3	0.42	11300	35	46	0.48	0.01
Jacket: Brass alloy (M90 PN-92/H-87025)	130	0.375	8940.9	112	505	0.42	0.01

E—Young’s modulus, ν—Poisson’s ratio, ρ—density.

**Table 5:** XCT parameters used in the experiment. Mean spectrum energy estimated using SpecCalc [52].

No.	Voltage	Current	Power	Mean Spectrum Energy	No Projections	Time	Voxel Size
	(kV)	(µA)	(W)	(keV)	(-)	(s)	(µm)
1	50	450	22.5	25.8	950	2	30.00
2	100	225	22.5	39.7			
3	150	150	22.5	51.6			
4	200	112	22.4	62.3			
